# Causes and Consequences of Hyperexcitation in Central Clock Neurons

**DOI:** 10.1371/journal.pcbi.1003196

**Published:** 2013-08-22

**Authors:** Casey O. Diekman, Mino D. C. Belle, Robert P. Irwin, Charles N. Allen, Hugh D. Piggins, Daniel B. Forger

**Affiliations:** 1 Department of Mathematical Sciences, New Jersey Institute of Technology, Newark, New Jersey, United States of America; 2 Faculty of Life Sciences, University of Manchester, Manchester, United Kingdom; 3 Center for Research on Occupational and Environmental Toxicology, Oregon Health & Science University, Portland, Oregon, United States of America; 4 Department of Mathematics and Department of Computational Medicine and Bioinformatics, University of Michigan, Ann Arbor, Michigan, United States of America; Indiana University, United States of America

## Abstract

Hyperexcited states, including depolarization block and depolarized low amplitude membrane oscillations (DLAMOs), have been observed in neurons of the suprachiasmatic nuclei (SCN), the site of the central mammalian circadian (∼24-hour) clock. The causes and consequences of this hyperexcitation have not yet been determined. Here, we explore how individual ionic currents contribute to these hyperexcited states, and how hyperexcitation can then influence molecular circadian timekeeping within SCN neurons. We developed a mathematical model of the electrical activity of SCN neurons, and experimentally verified its prediction that DLAMOs depend on post-synaptic L-type calcium current. The model predicts that hyperexcited states cause high intracellular calcium concentrations, which could trigger transcription of clock genes. The model also predicts that circadian control of certain ionic currents can induce hyperexcited states. Putting it all together into an integrative model, we show how membrane potential and calcium concentration provide a fast feedback that can enhance rhythmicity of the intracellular circadian clock. This work puts forward a novel role for electrical activity in circadian timekeeping, and suggests that hyperexcited states provide a general mechanism for linking membrane electrical dynamics to transcription activation in the nucleus.

## Author Summary

Daily rhythms in the behavior and physiology of mammals are coordinated by a group of neurons that constitute the central circadian (∼24-hour) clock. Clock neurons contain molecular feedback loops that lead to rhythmic expression of clock-related genes. Much progress has been made in the past two decades to understand the genetic basis of the molecular circadian clock. However, the relationship between the molecular clock and the primary output of clock neurons—their electrical activity—remains unclear. Here, we explore this relationship using computational modeling of an unusual electrical state that clock neurons enter at a certain time of day. We predict that this state causes high concentration of calcium ions inside clock neurons, which activates transcription of clock genes. We demonstrate that this additional feedback promotes 24-hour gene expression rhythms. Thus, we propose that electrical activity is not just an output of the clock, but also part of the core circadian timekeeping mechanism that plays an important role in health and disease.

## Introduction

The conventional theory of neuronal information processing is based on action potential (AP) firing [1], [2]. While signaling through APs is a ubiquitous form of neuronal communication throughout the nervous system, it is not the only mechanism through which neurons may signal. In particular, neurons that receive input that induces large inward currents (hyperexcitation) may display depolarization block, and be unable to fire APs due to voltage–gated sodium channel inactivation. For example, antipsychotic drugs can trigger depolarization block in midbrain dopamine neurons [3]–[5]. Depolarization block is also a feature of many mathematical models of neuronal dynamics [6], [7], including the canonical Hodgkin-Huxley [8] and Morris-Lecar [9] models. Furthermore, large inward currents can induce depolarized electrical states with low amplitude membrane oscillations (DLAMOs). Such depolarized states occur in intrinsically photosensitive retinal ganglion cells in the presence of bright light [10]. DLAMOs and depolarizing block occur spontaneously—not as the result of external stimulation—in neurons of the hypothalamic suprachiasmatic nuclei (SCN) [11]–[13]. Spontaneous depolarization block has also been reported in cerebellar nuclear neurons [14], [15]. These various depolarized states add complexity to the repertoire of neuronal communication.

In the SCN, which function as the central mammalian circadian (∼24-hour) pacemaker [16], depolarization block and DLAMOs occur only in a subset of SCN neurons and mostly during the latter half of the day [13]. Here, we study the ionic mechanisms that underlie these depolarized states in SCN neurons. We also seek to determine general principles for how neurons can spontaneously enter such states and the physiological role(s) they may play. Electrical activity of SCN neurons is not only important for sending timekeeping signals to other cells, but also for transmitting information from the external world to their intracellular molecular circadian clocks [17]. Thus, understanding the electrophysiology of SCN neurons is essential for understanding circadian timekeeping in mammals [18], and may also yield general insights into mechanisms for signaling from synapse to gene [19].

Our approach uses mathematical modeling in combination with experimental validation. We find that DLAMOs are caused by the interplay of L-type calcium and calcium-activated potassium (K_Ca_) currents. During depolarized states, we predict that intracellular calcium concentration reaches high (but physiological) levels. We propose that these daily elevated calcium levels activate clock gene transcription during the day, which in turn increases the expression of K_Ca_ and potassium leak currents to hyperpolarize the membrane at dusk and night. We show that this additional feedback loop between membrane excitability and gene expression can promote rhythmicity of the intracellular circadian clock.

## Results

### Model validation

**Figure 1:**
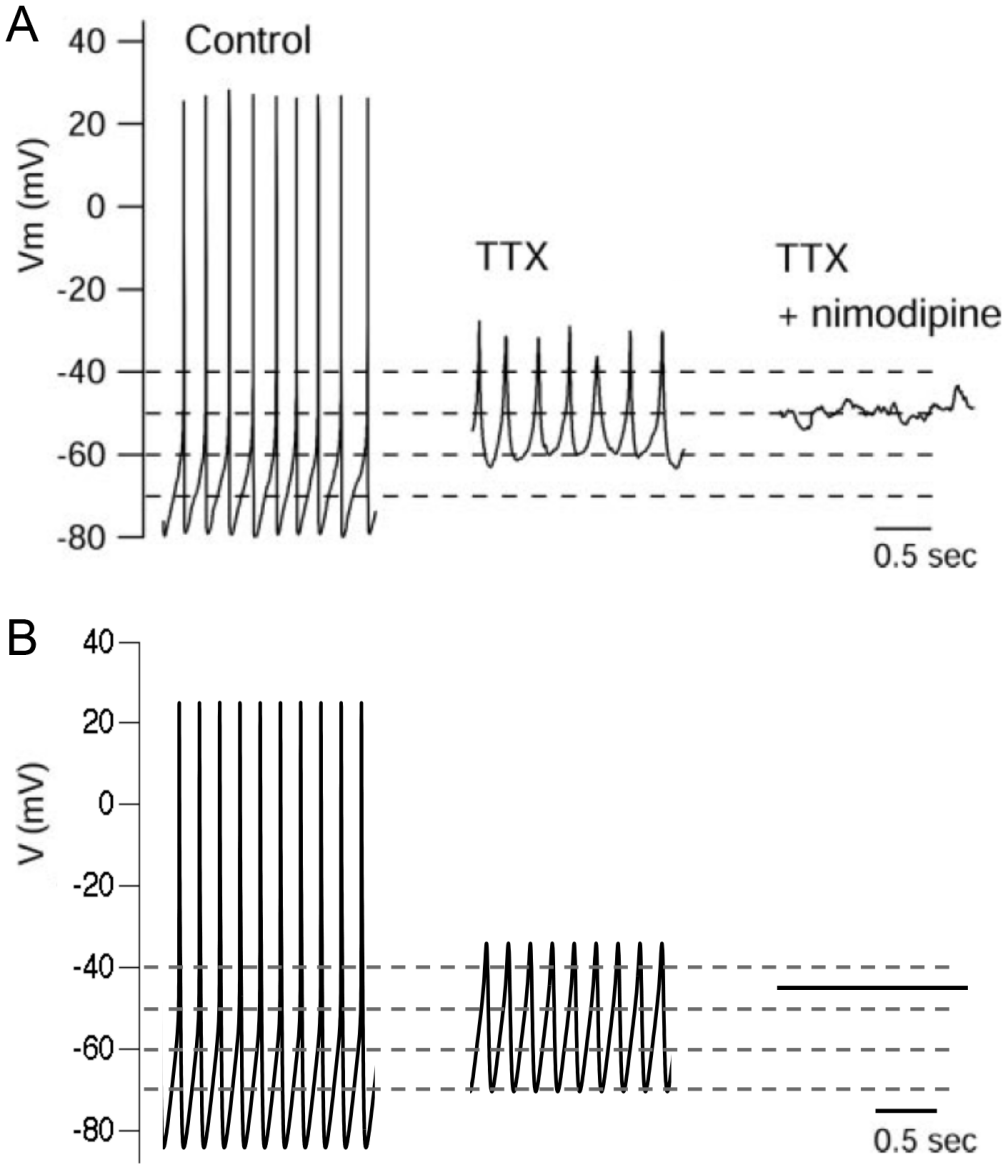
Model simulations of SCN AP firing and nimodipine-sensitive membrane oscillations in the presence of TTX match published experimental data. **A**. Reproduction of Figure 3B from Jackson et al. [24], with permission from Journal of Neuroscience, showing the effect of cumulative application of 300 nM TTX and 1 µM nimodipine on a spontaneously firing SCN neuron. The membrane potential oscillations in the presence of TTX (TTXLAMOs) are silenced by nimodipine. **B**. Model exhibiting spontaneous AP firing (*g_Na_* = 229 nS, *g_CaL_* = 6 nS) and membrane potential oscillations in the presence of simulated TTX (*g_Na_* = 0). The oscillations are silenced by simulated nimodipine application (*g_CaL_* = 0).

Our new computational model of a SCN neuron extends the model of Belle et al. [13] by incorporating a L-type calcium current, a K_Ca_ current, and intracellular calcium dynamics. The model is able to produce repetitive firing of APs in the absence of externally applied current (*I_app_* = 0), consistent with the spontaneous firing behavior of SCN neurons (see Brown and Piggins [18] for review). As in the original version of the model [20], the periodic solutions modeling repetitive AP firing arise when the quiescent steady state becomes unstable due to Hopf bifurcation [21]. The improved model more closely replicates the biophysical properties of SCN neurons as individual APs are now followed by an appropriate after hyperpolarization (AHP). The AHP amplitude is mediated by K_Ca_ currents and partly regulates the daily pattern of action potential firing frequency observed in SCN neurons [22], [23]. Another feature of SCN neurons are low amplitude membrane oscillations in the presence of TTX [11], [12]. Since these TTX-induced oscillations (TTXLAMOs) are seen in dissociated SCN cells [24], we concluded that they are intrinsic and unmasked by the post-synaptic effects of TTX. In the original version of the model [13], [20], simulation of the post-synaptic effects of TTX (setting sodium conductance (*g_Na_*) to 0) did not produce these oscillations, but instead resulted in a steady-state voltage (data not shown). In contrast, in our revised model, calcium and potassium currents interact to produce oscillations in the absence of sodium current (Figure 1B), consistent with the TTXLAMOs previously reported in the literature (Figure 1A). Additionally, in agreement with experimental data, simulating the application of the L-type calcium channel blocker nimodipine abolished TTXLAMOs (Figure 1A,B). An interesting feature seen both in the model and experimental data was that blocking calcium channels leads to a depolarization rather than a hyperpolarization of SCN cells. This results from a reduction in the amplitude of K_Ca_ currents, an important parameter that determines the resting membrane potential (RMP) of some SCN neurons [13].

### Ionic mechanisms of depolarized membrane oscillations

**Figure 2:**
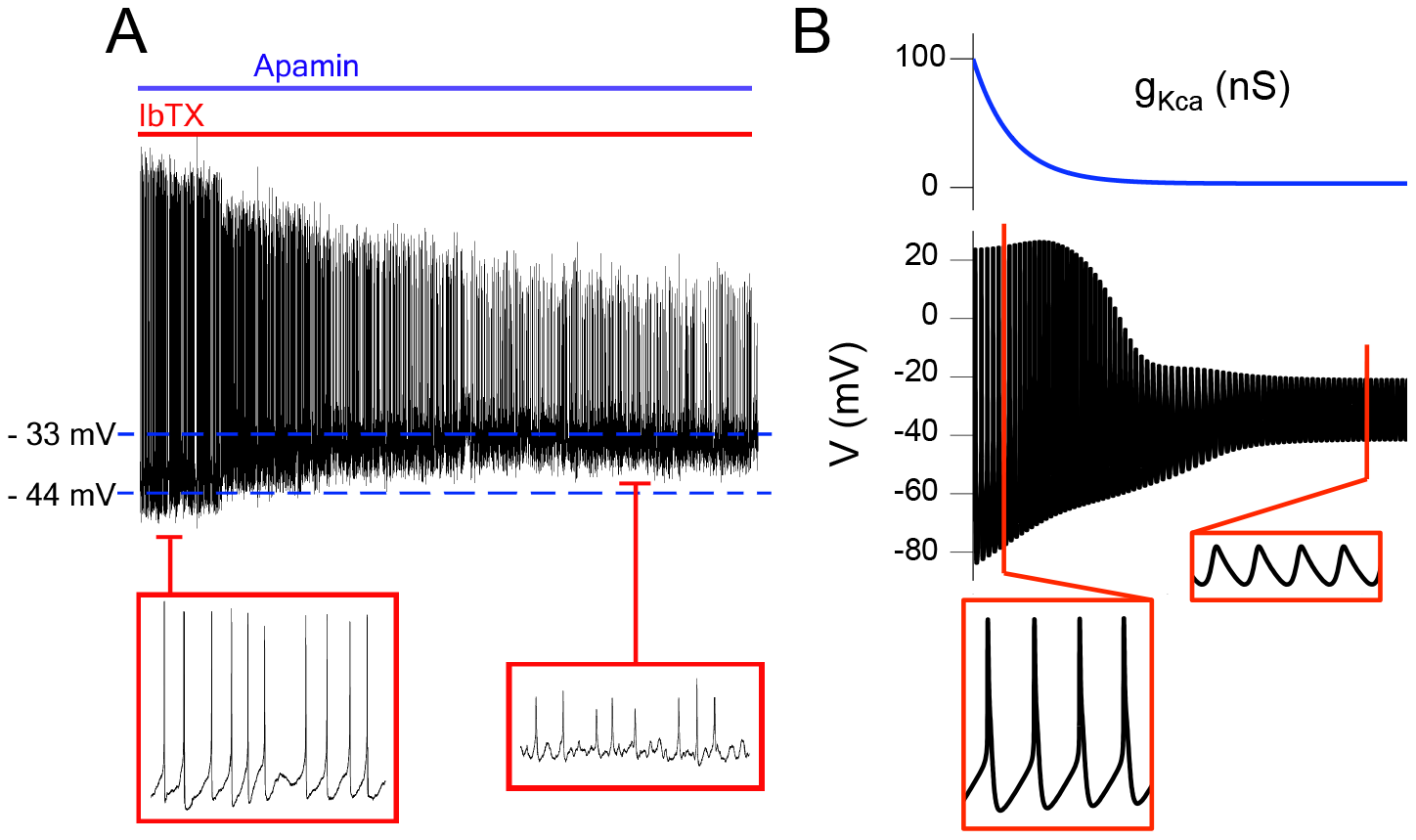
Model simulations of SCN membrane depolarization due to reduced K_Ca_ currents match published experimental data. **A**. Concomitant application of K_Ca_ channel blockers iberiotoxin (IbTX, 100 nM) and apamin (200 nM) on a spontaneously firing *Per1*::eGFP+*ve* neuron. The membrane depolarizes and the cell displays DLAMOs, as in Figure 4A of Belle et al. [13]. **B**. Model transitioning from AP firing to DLAMOs as *g_KCa_* is gradually reduced.

A subpopulation of SCN neurons, specifically those expressing detectable levels of the *Period1* gene (*Per1*::eGFP+*ve* neurons), are at a depolarized RMP during much of the afternoon [13], and can show spontaneous DLAMOs. In our model, reducing K_Ca_ conductance (*g_KCa_*) can depolarize the membrane and transition a cell from generating APs to DLAMOs (Figure 2B), consistent with the effect of K_Ca_ channel blockers on SCN neurons *in vitro* (Figure 2A). We hypothesized that DLAMOs occur through a similar mechanism to TTXLAMOs. According to this hypothesis, calcium currents would be larger than sodium currents in the depolarized states since most of the TTX sensitive sodium channels will be inactivated. Thus, we expected that application of TTX would have little effect on DLAMOs, whereas application of nimodipine would inhibit these oscillations [12].

**Figure 3:**
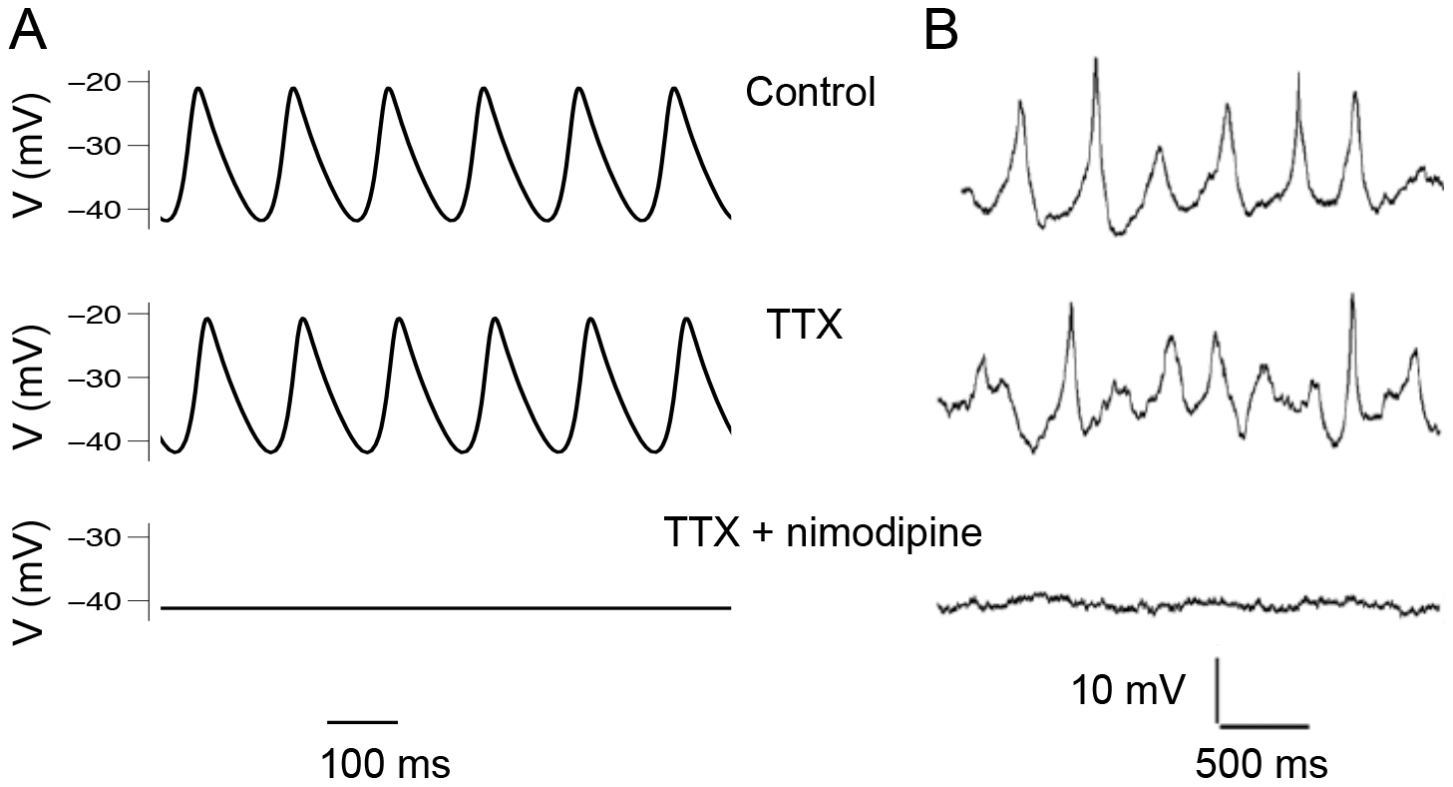
Role of L-type Ca^2+^ and TTX-sensitive Na^+^ channels in mediating spontaneous DLAMOs. **A**. Model exhibiting spontaneous DLAMOs (Control: *g_Na_* = 229 nS, *g_CaL_* = 6 nS, *g_KCa_* = 3 nS, RMP of −31 mV), which persist in simulated TTX (*g_Na_* = 0), but are abolished by simulated nimodipine (*g_CaL_* = 0 nS). **B**. Recording from a *Per1*::eGFP*+ve* neuron exhibiting spontaneous DLAMOs (Control: RMP of −34 mV). DLAMOs persist in 1 µM TTX but were reversibly abolished by 2 µM nimodipine. During TTX and nimodipine application, the RMP was actively maintained at −34 mV to prevent membrane depolarization and inactivation of L-type Ca^2+^ channels.

We tested this hypothesis by simulating a neuron in a state producing spontaneous DLAMOs (Figure 3A). When the post-synaptic effect of TTX application was simulated (sodium conductance (*g_Na_*) set to 0), in neurons that otherwise would show DLAMOs, very little change in the neuron's behavior was seen. However, simulation of nimodipine application (L-type calcium conductance (*g_CaL_*) set to 0) abolished all oscillations. To test this experimentally, we recorded from 42 *Per1*::eGFP+*ve* neurons that spontaneously exhibited DLAMOs during the projected day. Validating the model's predictions, we find that application of TTX had only a subtle effect on the oscillations, while nimodipine application abolished them (Figure 3B). This provides pharmacological evidence that DLAMOs require L-type calcium current, similar to the membrane oscillations seen in the presence of TTX [12], [24].

The fact that DLAMOs require L-type calcium current suggests that they may be sensitive to factors affecting calcium homeostasis in SCN neurons, since inactivation of L-type current is primarily calcium-dependent [25]. To explore this using our model, we simultaneously varied *g_KCa_* and the basal calcium concentration near the membrane (*b_s_*). Mathematically, the transition to DLAMOs occurs through a Hopf bifurcation from a depolarized steady state. For a given basal calcium concentration, DLAMOs are seen once *g_KCa_* exceeds a minimal value (Figure S1). As basal calcium is increased, less *g_KCa_* is required for DLAMOs to be seen (Figure S2). However, if *g_KCa_* is too small, then a Hopf bifurcation does not occur—increasing *b_s_* instead leads to saddle-node bifurcation and a transition from the depolarized steady state to a hyperpolarized steady state (Figures S3 and S4). Thus, the onset of DLAMOs in a population of SCN neurons may be heterogeneous, in accordance with heterogeneity in the balance of calcium homeostasis and K_Ca_ channel expression across cells. These observations lead to the following testable predictions. If a SCN neuron is in a depolarized steady state, raising extracellular Ca^2+^ to a sufficient level may induce DLAMOs. Once the cell is exhibiting DLAMOs, raising extracellular Ca^2+^ further will induce a hyperpolarized steady state. If elevating extracellular Ca^2+^ does not have the predicted effects, it is an indication that K_Ca_ channel expression is very low in that cell. In such a cell, application of a synthetic K_Ca_ channel opener such as NS004 or NS11021 [26] should initiate DLAMOs.

**Figure 4:**
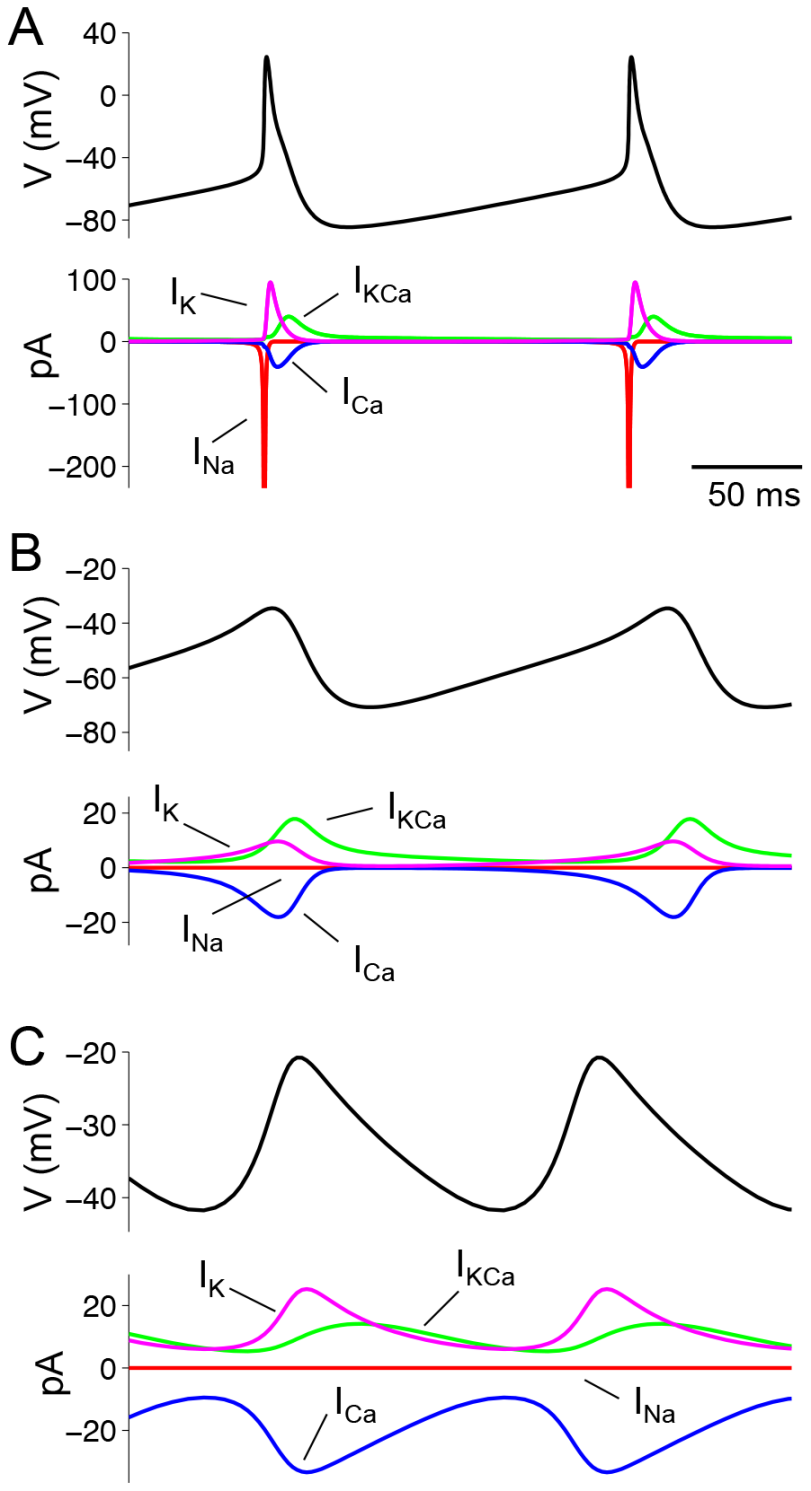
Ionic mechanisms of SCN electrical behaviors. **A**. *I_Na_*, *I_K_*, *I_Ca_* and *I_KCa_* during a simulated AP (peak of *I_Na_* current (∼450 pA) not shown). **B–C**. Low amplitude oscillations in absence of sodium current due to (**B**) simulated TTX application (*g_Na_* = 0) or (**C**) inactivation at depolarized membrane potential (*g_KCa_* = 3 nS). Although the oscillations in (**B**) and (**C**) are both produced by the interplay between *I_Ca_* and potassium currents, the mean level of the currents are greater in (**C**).

To better understand the ionic currents underlying the electrical behaviors of SCN neurons, we first considered the contribution of sodium (*I_Na_*), calcium (*I_Ca_*), potassium (*I_K_*) and calcium-activated potassium (*I_KCa_*) currents during simulated AP firing (Figure 4A). The model AP is characterized by rapid activation of *I_Na_*, quickly followed by activation of *I_K_* and inactivation of *I_Na_*. Likewise, activation of *I_KCa_* follows activation of *I_Ca_*, but these currents are slower and lower in magnitude. Since the simulated ionic currents closely resembled the currents measured during AP clamp experiments [24], we then used the model to predict the ionic contributions driving the low amplitude membrane oscillations observed in SCN neurons. We found that both TTXLAMOs and DLAMOs occurred via a balance between calcium, potassium, and calcium-activated potassium currents (Figure 4B,C). Calcium current activation preceded the opening of potassium currents and dominated the rising phase of the oscillations. As calcium enters the cell it activates K_Ca_ channels, which contribute to the falling phase of the oscillations. In simulations of TTXLAMOs, sodium currents were set to zero to reflect the post-synaptic effect of TTX. In DLAMOs, the sodium current was naturally minimal due to the depolarized state of the neuron.

### Prediction of intracellular calcium levels

**Figure 5:**
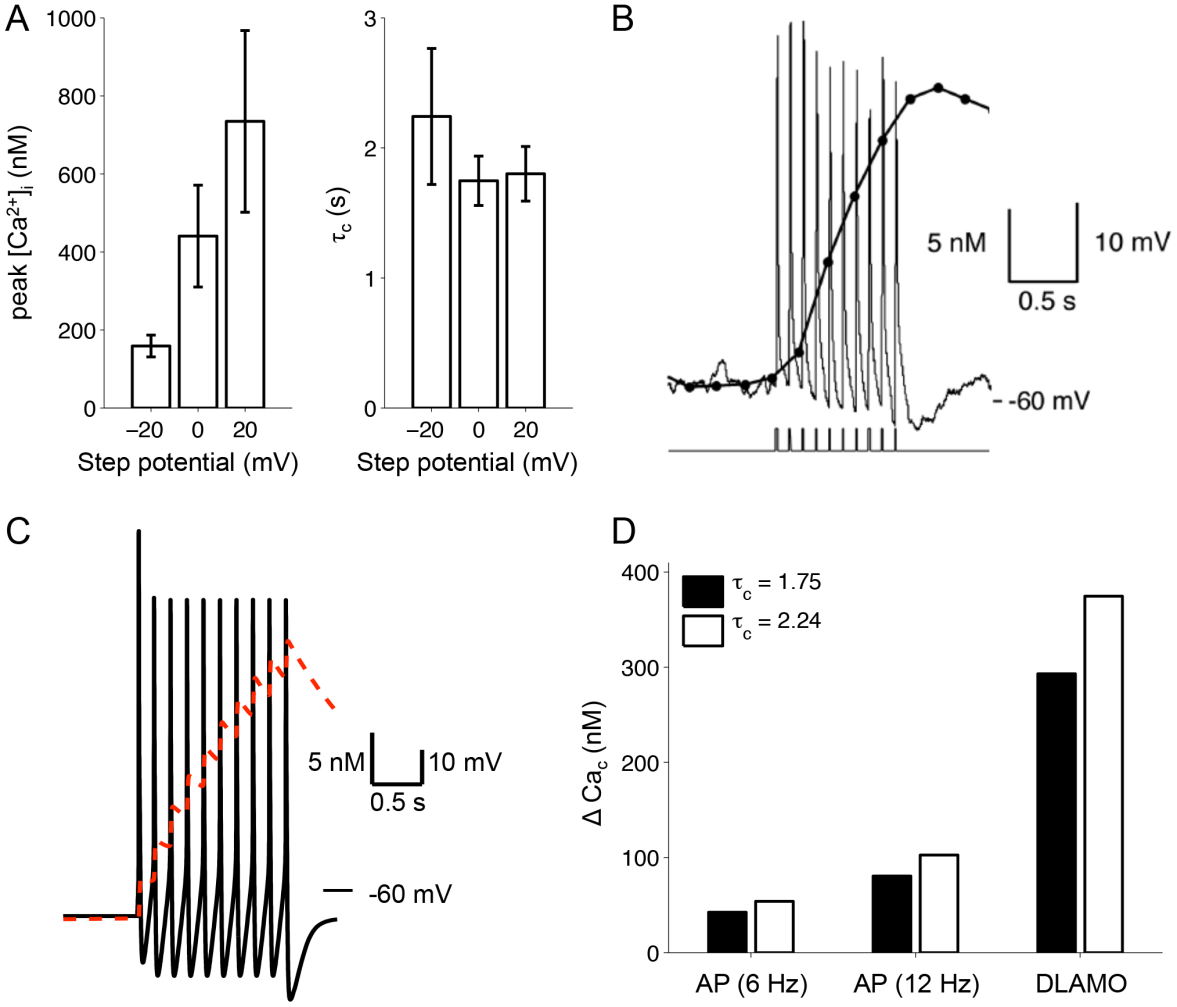
Measurement of calcium clearance rate and prediction of intracellular calcium levels. **A**. The mean peak calcium concentration (left) and calcium decay time constant (right) measured in SCN neurons that were held at −60 mV and then briefly (200 ms) depolarized to membrane potentials of −20, 0, and +20 mV (n = 10 cells, mean ± SEM). **B**. Reproduction of Figure 6E from [27], with permission from Journal of Neuroscience, showing the change in [Ca^2+^]_i_ in response to depolarizing pulses (100 pA, 10 ms) in a current-clamped SCN neuron. **C**. Rise and fall of *Ca_c_* evoked by a train of spontaneous APs in the model upon removal of a hyperpolarizing current (*I_app_* = −5 pA). **D**. The model predicts a much higher level of calcium accumulation during DLAMOs than during AP firing.

**Figure 6:**
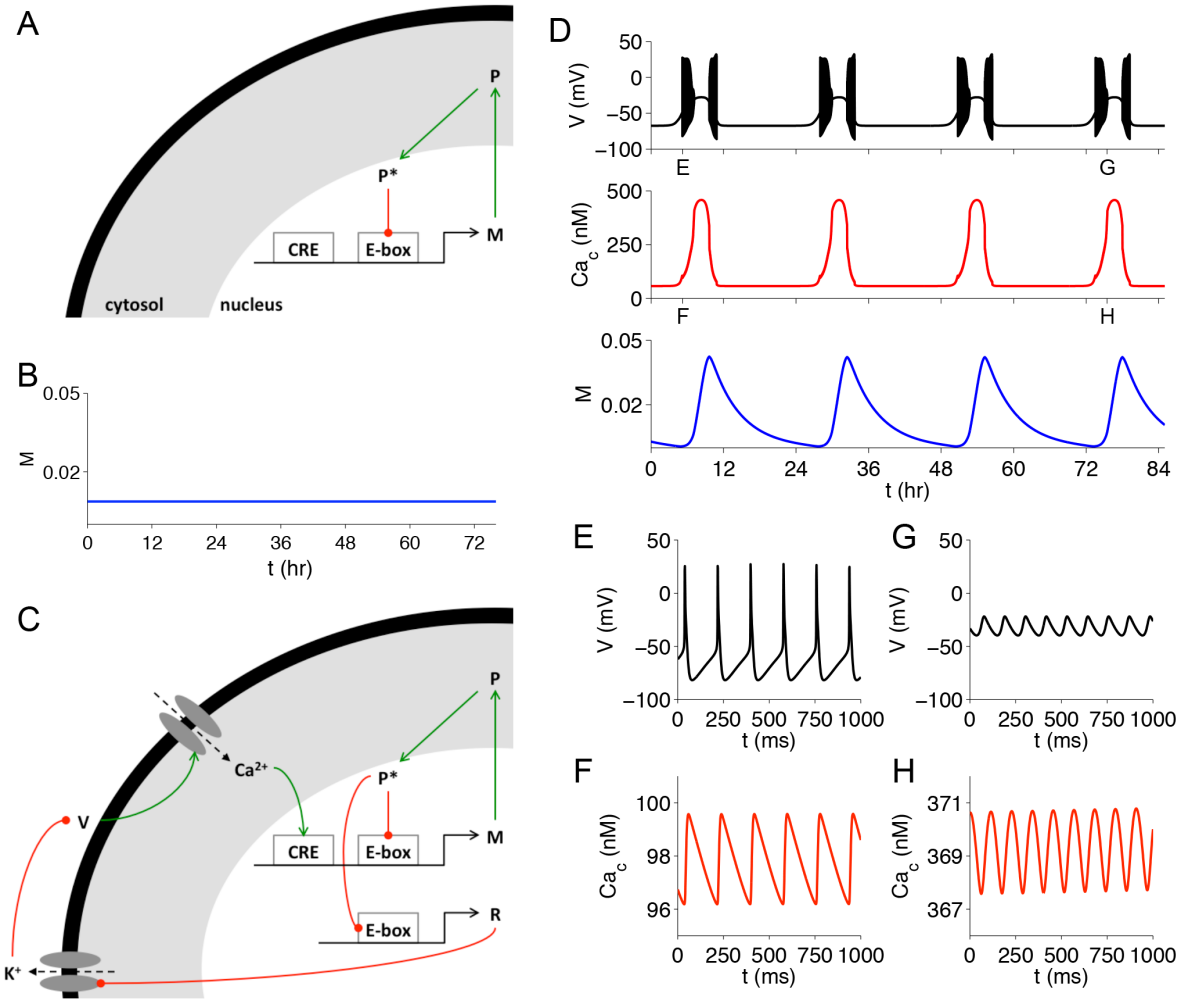
Hyperexcitation enhances gene expression rhythms. **A**. Schematic of a simple model of gene regulation with negative feedback. A clock gene's protein product (*P*) is phosphorylated (P*), and then binds to a promoter sequence (E-box) and inhibits production of its own mRNA (*M*). **B**. Simulation of the model in (**A**) showing that the level of mRNA (*M*) does not oscillate. **C**. Extension of model in (**A**) to incorporate membrane excitability. The mRNA of another gene (*R*), under control of the same E-box as the clock gene, reduces *g_KCa_* and *g_K-leak_*, which depolarizes the membrane (*V*), causing more calcium to enter the cell and activating transcription of *M* through the CRE. **D**. Simulation of the model in (**C**) showing that inclusion of membrane excitability induces ∼24-hour oscillations in clock gene expression. The hyperexcited states (top) lead to peaks in calcium concentration (middle) and mRNA level (bottom). **E–H**. One-second segments of voltage and cytosolic calcium traces from (**D**) during AP firing (**E**,**F**) and DLAMOs (**G**,**H**).

A key difference between TTXLAMOs and DLAMOs was the mean calcium current. In TTXLAMOs, the mean calcium current was small and at times near zero (Figure 4B). However, in DLAMOs, the mean (and minimal) calcium current was substantially different from zero (Figure 4C). We sought to predict the effect of this increased calcium influx on intracellular calcium levels. Our minimal model for intracellular calcium (*Ca_c_*) dynamics (see Materials and Methods) requires the estimation of two parameters: a clearance rate (1/τ*_c_*) and a factor for converting calcium current to concentration (*k_c_*). Since the intracellular calcium concentration ([Ca^2+^]_i_) is typically tightly regulated, we hypothesized that in order to avoid accumulation of calcium ions in the cytosol, the rate of calcium clearance would be higher at depolarized membrane potentials. To test this hypothesis, we measured [Ca^2+^]_i_ in ten SCN neurons following 200 ms duration depolarizing voltage steps from a holding potential of −60 mV, and estimated τ*_c_* by fitting [Ca^2+^]_i_ to an exponential decay. The means of the peak [Ca^2+^]_i_ after voltage steps to −20 mV (158.9±28.0 nM, mean ± SEM), 0 mV (440.8±130.5 nM), and +20 mV (734.8±232.9 nM) were not equal (one-way ANOVA, *p* = 0.046), and peak [Ca^2+^]_i_ was significantly higher in the +20 mV group than in the −20 mV group (Tukey's HSD test, α = 0.05) (Figure 5A, left). However, we found no significant differences in τ*_c_* between any of the three voltage steps (2.24±0.52 s, 1.75±0.19 s, 1.80±0.21 s for the −20, 0, and +20 mV groups respectively, one-way ANOVA, *p* = 0.543) (Figure 5A, right). We then set τ*_c_* = 2.24 s, and chose *k_c_* based on previously reported measurements of Δ[Ca^2+^]_i_ in SCN neurons evoked by a series of brief depolarizing pulses (Figure 6E of Irwin and Allen [27]) and during AP firing (Figure 4C of Irwin and Allen [27]). Figure 5C shows that our model produces similar changes in intracellular calcium during a train of spontaneous APs as seen in the experimental data (Figure 5B).

Next, we compared the intracellular calcium levels predicted by the model during quiescence (−65 mV RMP), extended AP firing, and DLAMOs. The model predicts that DLAMOs induce a much greater increase in steady-state intracellular calcium (Δ*Ca_c_*>290 nM) than AP firing at 6 Hz (Δ*Ca_c_*<55 nM) and 12 Hz (Δ*Ca_c_*<105 nM) (Figure 5D). Although intracellular calcium levels in hyperexcited clock neurons have yet to be reported, this prediction is consistent with the findings of two related studies. Firstly, Irwin and Allen [27] show that [Ca^2+^]_i_ increases exponentially with increased membrane potential (ranging from −80 to −40 mV) in SCN neurons. Their data suggest that voltage steps to depolarized membrane potentials increase intracellular calcium more than firing action potentials for a similar duration. Secondly, Pennartz et al. [12] measured inward calcium currents in SCN neurons during sustained (>300 ms) depolarizing voltage steps. At −30 mV, the calcium current appears to be nearing a steady state of 40 pA or greater. This calcium current is larger than the calcium current predicted by our simulations during the peak of a DLAMO (33 pA). Taken together, these data provide strong, albeit indirect, evidence supporting our prediction of elevated intracellular calcium levels during DLAMOs.

### Hyperexcitation enhances rhythmicity of intracellular circadian clock

Elevation of intracellular calcium levels may play an important role in the rhythmic gene expression that constitutes the molecular circadian clocks within SCN neurons. A major phase-shifting and entrainment pathway for these clocks involves CREB-dependent activation of *Per1* and *Per2* transcription [28]. Dolmetsch et al. [29] reported that for CREB activation in cortical neurons, [Ca^2+^]_i_ levels in excess of 400 nM are required. In our simulations, such high calcium levels are achieved only during hyperexcited states.

To explore the relationship between hyperexcitation and the intracellular circadian clock, we integrated our model of SCN neuron excitability with a simple model of gene regulation based on the Goodwin oscillator [30]. In this model, a clock gene is transcribed into mRNA (*M*), the mRNA is translated into protein (*P*), the protein is phosphorylated (P*), and the phosphorylated protein binds to an enhancer (E-box) in the promoter region of the clock gene, inhibiting its transcription (see Figure 6A for a model schematic and Materials and Methods for the model equations). This negative feedback loop can lead to oscillations if one assumes positive cooperativity among a large number of molecules (*n*>8) in repression of the E-box [31]. Here, we set *n* = 4, and show through simulation that this model does not produce oscillations with the chosen parameters (Figure 6B). We then extended the model to incorporate SCN membrane excitability as diagrammed in Figure 6C. We assume there is another gene product (*R*) under the control of the same E-box as the clock gene, and that *R* downregulates the activity of potassium channels (specifically K_Ca_ and potassium leak currents) in the membrane. This is motivated by known circadian rhythms in the expression of these channels ([32]–[34], see Colwell [35] for review). These currents hyperpolarize the membrane potential (*V*), which closes voltage-gated calcium channels and reduces the inward calcium current. This affects intracellular calcium concentration (*Ca_c_*), which regulates clock gene transcription through cAMP response elements (CREs). The extended model produces ∼24-hour oscillations in *M* (Figure 6D) using the same set of parameters (including *n* = 4) that did not produce oscillations in the basic gene regulation model (Figure 6B). The oscillations are enabled by the additional feedback present in the extended model: since the phosphorylated clock protein inhibits *R*, increasing P* leads to an up-regulation of potassium currents, membrane hyperpolarization, and less intracellular calcium, thus ultimately decreasing clock gene transcription. On the other hand, decreasing P* leads to down-regulation of potassium currents, membrane depolarization, and more intracellular calcium, thereby increasing clock gene transcription. These results suggest that electrical activity, and in particular hyperexcited states, are more than just an output signal of the intracellular clock and actually play a key role in rhythm generation.

We also note that the proposed mechanism of signaling from membrane to gene transcription within a single cell via depolarized states does not necessarily require AP firing. In simulations of our extended model, ∼24-hour oscillations in cytosolic calcium and gene expression persist in the presence of TTX (Figure S5). This result emphasizes the role of a cell's membrane potential, more so than its firing rate, as a regulator of its intracellular molecular clock.

## Discussion

Previous studies have shown higher levels of *Per1* expression in the SCN [36], [37] during the time of day when RMPs are more depolarized [38]. In this study we provide evidence that the depolarized RMPs reported in Belle et al. [13] correspond with high levels of steady-state intracellular calcium concentration that may be needed for activation of the *Per1* gene through CREB [39], [40]. This is in line with Irwin and Allen [27], who show an increase in calcium levels in response to depolarization of SCN neurons, as well as Dolmetsch et al. [29], who show that high calcium levels are needed for CREB signaling. Further experimental work is needed to validate our predictions, including measurement of intracellular calcium levels in spontaneously depolarized SCN neurons and the specific levels of calcium needed to activate CREB signaling in these neurons.

While the basic mechanism of circadian timekeeping in mammalian cells is a transcriptional-translational negative feedback loop, electrical activity has sometimes been considered part of the core timekeeping mechanism [41]–[43]. Our work generates the hypothesis that depolarized states in clock neurons are part of the intracellular timekeeping mechanism. Accordingly then, the molecular clock controls the transcription of potassium channels, which, when expressed, could take the electrical state of the neuron into and out of depolarization block or DLAMOs. Depolarized states trigger high calcium, which in turn triggers the transcription of *Per1* and *Per2*.

This hypothesis is consistent with and extends previously published data. In cerebellar granule cell cultures, *Per1* expression was found to be dependent on the depolarization state of the neuron, and prevention of Ca^2+^ influx (by pharmacological blocking of voltage-gated calcium channels) reduced *mPer1* induction [44]. Remarkably, cerebellar granule neurons also exhibit a spontaneous depolarized state at RMPs of −28 to 34 mV [14], [15]. In *Drosophila*, electrical silencing of pacemaker neurons stops the molecular clock [45], [46]. In SCN neurons, inhibiting Ca^2+^ influx [17] or CREB signaling [47] interferes with circadian rhythm generation. Here, we propose that in SCN neurons, calcium-activated potassium currents are diminished during the day, leading to higher calcium levels and more expression of PER1/PER2 at the expected phase. In addition, CREB is known to positively regulate large-conductance K_Ca_ (BK) channel expression in *Drosophila* [48], providing another potential mechanism for feedback.

Mathematical modeling is an established tool for understanding the complex interaction of neuronal ion channels and calcium dynamics [49], [50]. Several models of SCN neurons and circadian clocks contain calcium as a key component [51]–[55]. Our model is the first to estimate calcium levels in hyperexcited SCN neurons and compare such estimates with experimental data on cytosolic calcium levels, as well as the increases in intracellular calcium from action potentials. We relied on measurements of the overall cytosolic calcium level, and found a simple exponential clearance was sufficient to reproduce our data. When further experimental data on calcium regulation (e.g. release from intracellular stores) in SCN neurons becomes available, these details should be incorporated into our mathematical models. In our model, we have focused on the effect of cytosolic calcium on *K_Ca_* and potassium leak currents, based on previous reports of rhythmic regulation of these channels [32]–[34]. However, there is evidence that the circadian clock may also regulate A-type potassium and L-type calcium channels [12], [56], and the effects of this additional control could be considered in future models.

Several experimental studies have used TTX to assess the role of sodium-dependent APs on circadian rhythmicity. Infusion of TTX into the SCN of freely moving rats disrupts behavioral rhythms but not internal circadian timekeeping [57]. *In vitro* application of TTX that abolishes AP firing in rat and hamster SCN slices does not affect the rhythm in SCN metabolism [58]. TTX also eliminates firing in SCN cultures [59], but does not block the cytosolic calcium rhythm [60]. Finally, single-cell bioluminescence measurements in SCN slice cultures indicate that TTX-sensitive APs are required for both robust clock gene expression rhythms in individual neurons and synchronization of these rhythms across cells [61]. Our simulation results (Figure S5) suggest that TTX dampens clock gene expression rhythms measured grossly across a population of SCN neurons primarily through accumulation of desynchronization rather than damping of individual oscillators. These simulations also predict that TTXLAMOs would only occur at certain times of day, which may help explain why these types of oscillations have not been widely reported in the literature outside of their initial discovery [12], [24].

Mizrak et al. [62] report that hyperexciting *Drosophila* clock neurons creates a morning-like expression profile for many circadian genes, while hyperpolarizing them creates an evening-like transcriptome. This underscores the different nature of neuronal signaling in depolarized versus conventional states. We predict that depolarized states in SCN neurons trigger transcription activation and can enhance rhythmicity, but future work is needed to understand how these depolarized states affect the dynamics of intracellular timekeeping in detailed mammalian clock models [63] and their other implications for neuronal information processing.

## Materials and Methods

### Mathematical modeling

We extended the computational model of a SCN neuron from Belle et al. [13] to include L-type calcium current (*I_CaL_*), calcium-activated potassium current (*I_KCa_*), and intracellular calcium dynamics. The current balance equation for the revised model is:
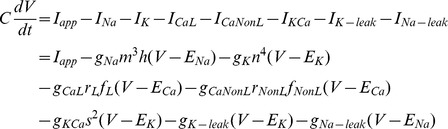
where *C* = 5.7 pF, *I_app_* = 0 pA, *g_Na_* = 229 nS, *g_K_* = 3 nS, *g_CaL_* = 6 nS, *g_CaNonL_* = 20 nS, *g_KCa_* = 100 nS, *g_K-leak_* = 0.0333 nS, *g_Na-leak_* = 0.0576 nS, *E_Na_* = 45 mV, *E_K_* = −97 mV, and *E_Ca_* = 54 mV unless specified otherwise.

The dynamics of the gating variables *q* = *m*, *h*, *n*, *r_L_, r_NonL_, f_NonL_*, and *s* are:
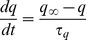


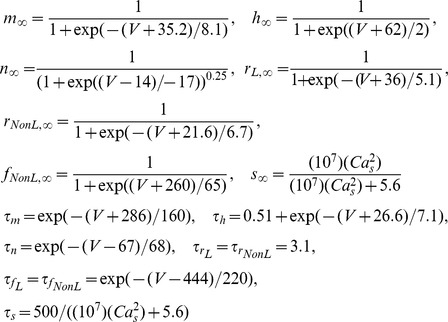
The L-type calcium current model is based on measurements of nimodipine-sensitive current in SCN neurons [24]. Since inactivation of L-type current is primarily calcium-dependent, we modeled it as:
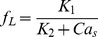
where *K_1_* = 3.93E-5 and *K_2_* = 6.55E-4 mM.

The *I_KCa_* kinetics follow the form of the voltage-independent calcium-dependent potassium current given in Yamada et al. [25], with parameter values chosen based on measurements of total K_Ca_ current in SCN neurons during action potential clamp experiments [24].

Intracellular calcium dynamics are extremely complex and involve many different mechanisms, such as buffering, uptake into and release from intracellular stores, and extrusion through membrane pumps. However, because in SCN neurons many of the details of these mechanisms have not been measured experimentally, we chose to use a very simple model of calcium dynamics that could be fitted directly to experimental data from these neurons. Our model represents all calcium handling mechanisms with a single term for the removal of free calcium ions from the cytosol, as in Booth et al. [64]. In our model, calcium enters the cytosol through voltage-gated calcium channels only; we do not explicitly consider release from intracellular stores. Thus, the concentration of free intracellular calcium ions is determined by the following equation:
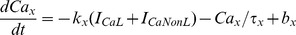
and is tracked separately in two compartments: one representing a thin spherical shell near the membrane surface where the binding of intracellular calcium ions to K_Ca_ channels occurs (*Ca_s_*), and the other representing the entire cytosol (*Ca_c_*). The parameter *k* converts Ca^2+^ current (pA) to Ca^2+^ concentration (mM), and τ is the Ca^2+^ clearance time constant. We set *k_s_* = 1.65e-4 mM/fC, corresponding to an SCN cell with a radius of about 5 µm [65] and a shell depth of 0.1 µm. This depth is a common choice for models where [Ca^2+^]_i_ is relevant for K_Ca_ channel activation [25], [66]. Unless specified otherwise, we set *k_c_* = 8.59e-9 mM/fC, *τ_c_* = 1.75e3 ms, and *τ_s_* = 0.1 ms to match measurements of the total calcium current entering an SCN neuron during AP clamp from Jackson et al. [24], and our own measurements of [Ca^2+^]_i_ in SCN neurons (see Figure 5). The constant term *b* sets the basal level of calcium in the absence of spiking, we chose *b_c_* = 3.1e-8 mM/ms (and *b*_s_ = 5.425e-4 mM/ms) so that the steady-state values of *Ca_c_* and *Ca_s_* are approximately 54 nM in the absence of calcium entry (*I_CaL_* = *I_CaNonL_* = 0). See Figure S6 for a visualization of the evolution of *Ca_c_* and *Ca_s_* during AP firing.

Our basic model of gene regulation (Figure 6A) assumes linear degradation of mRNA (*M*), protein (*P*), and phosphorylated protein (P*) and has the form:
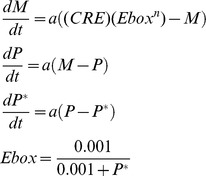
The parameter *a* scales time, and was set to 5.6E-8 ms^−1^.

The extended gene regulation model (Figure 6C) incorporates membrane excitability by making the maximal conductances of calcium-activated potassium (*g_KCa_*) and potassium leak (*g_K-leak_*) dependent on the activity of the E-box:
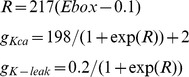



In both the basic gene regulation model and the extended version, we assume that the CRE and the E-box interact multiplicatively [67]. In the basic model, the CRE activity is set to a constant value, *CRE* = 77.3, whereas in the extended model it tracks calcium concentration:
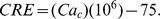



All differential equations are expressed in millisecond time units and all simulations were performed using the *ode15s* and *ode23tb* routines in Matlab® 2008 (Mathworks, Natick, MA). The variables *M*, *P*, and P^*^ were assigned initial conditions of 0.1, and all other state variables were initialized to zero. Bifurcation diagrams were computed using XPPAUT [68].

### Current-clamp recordings

We carried out targeted whole-cell electrophysiology in SCN neurons from fourteen male and female mice (∼2–3 months old) heterozygous for *Per1*::d2EGFP transgene (*Per1*::eGFP-expressing mice: a gift from D. McMahon, Vanderbilt University, TN, USA) bred and supplied by the Biological Services Facility of the University of Manchester. In these animals, a destabilized form of enhanced green fluorescent protein (eGFP) reports the activity of the *mPer1* promoter [69]. Animal housing, mid-coronal SCN brain slice preparation, current-clamp recordings, and *Per1*::eGFP neuron visualization were performed as described in Belle et al. [13]. Drugs were bath applied in artificial cerebro-spinal fluid (aCSF) delivered to the slice by gravity feed. Stock solutions for nimodipine (Tocris, Bristol, UK) were prepared by dissolving in Dimethyl sulfoxide (DMSO): final working concentration of DMSO did not exceed 0.01%. Tetrodotoxin (TTX: Tocris) was dissolved in aCSF. All experimental procedures were carried out according to the provisions of the UK Animal (Scientific Procedures) Act 1986.

### Calcium imaging

Simultaneous electrophysiological recordings and calcium imaging were performed using three male C57BL/6 mice (heterozygous for *Per1*::Venus expression, a gift from K. Obrietan, Ohio State University, OH, USA) that were housed for at least 1 week on a 12 : 12 h light : dark cycle. During the light phase, 7–12 week-old mice were anesthetized with isofluorane (Novaplus, UK), their brains removed and coronal hypothalamic slices (200–225 µm) containing the SCN were cut with a vibrating blade microtome (Leica-Microsystems VT1000S; Wetzlar, Germany). The tissue was surrounded by ice-cold artificial cerebrospinal fluid (ACSF) containing (in mM): NaCl, 126; KCl, 2.5; NaH_2_PO_4_, 1.2; MgCl_2_, 4; CaCl_2_, 0.5; glucose, 11; NaHCO_3_, 26; and saturated with 5% CO_2_ and 95% O_2_. The slices were maintained in a recording chamber (36°C) with a continuous laminar flow (1–2 mL/min) of an aCSF solution consisting of (in mM): NaCl, 132.5; KCl, 2.5; NaH_2_PO_4_, 1.2; MgCl_2_, 1.2; CaCl_2_, 2.4; glucose, 11; NaHCO_3_, 22; and bubbled with 5% CO_2_ and 95% O_2_. Whole-cell patch-clamp recordings of SCN neurons were performed during the night phase 1–8 h after slice preparation. Microelectrodes 5–10 MΩ were filled with an internal solution containing (in mM, Sigma-Aldrich, St. Louis, MO, USA): K-gluconate, 130; KCl, 5; HEPES, 10; MgATP 4; TrisGTP 0.4; ,di(tris) phosphocreatine 10; adjusted to pH 7.3 with KOH at (280–300 mOsm) to which was added bis-fura-2 hexapotassium salt (50 µm) (Molecular Probes-Life Technologies, Grand Island, NY, USA). After making a 1–5 GΩ membrane seal, the neuron was brought into whole-cell mode and rapidly filled with bis-fura-2. A HEKA EPC9 amplifier (HEKA, Lambrecht, Germany) and PatchMaster (HEKA) software were used to hold the neuron at a membrane potential of −60 mV and step (200 ms) the membrane potential to −20, 0, or 20 mV at 30–60 sec intervals to allow for recovery of the ensuing Ca^2+^ transient. While in voltage-clamp, 4 of the 10 neurons tested had TTX (0.5 µM) present but demonstrated no difference in the voltage step-induced change in [Ca^2+^]_i_ (steps −20, 0 and 20 mV respectively, *p* = 0.50, *p* = 0.68, *p* = 0.56; unpaired two-tail *t*-test). Recordings were performed within 10 min of entering whole-cell mode. Calcium measurements were obtained and converted to estimated [Ca^2+^]_i_ values as previously reported [27], [70]. The Institutional Animal Care and Use Committee of OHSU approved, in advance, all procedures involving animals.

## Supporting Information

Figure S1 One-parameter bifurcation diagram of the model's behavior as a function of K_Ca_ conductance. For a given basal calcium concentration in the shell (here *b_s_* = 5.425e-4 mM/ms), the depolarized steady state (solid black line) becomes unstable (dashed black line) through supercritical Hopf bifurcation (HB at *g_KCa_* = 2.82 nS, *V* = −30.8 mV) as *g_KCa_* is increased. At the HB point, stable periodic solutions modeling DLAMOs are born (circles indicate maximum and minimum voltage values for the oscillations at various *g_KCa_* values). The regions of *g_KCa_* corresponding to the depolarized steady state and to DLAMOS are shaded light red and light green respectively. The depolarized steady state persists for *g_KCa_* values below 2.75 nS.
(TIF)

Figure S2 Two-parameter bifurcation diagram showing the location of the Hopf bifurcation point in Figure S1 (blue line) as a function of *g_KCa_* and basal calcium concentration in the shell. As *b_s_* increases, less K_Ca_ conductance is required to undergo bifurcation from the depolarized steady state to DLAMOs. A second Hopf bifurcation (red line) from a hyperpolarized steady state occurs at certain combinations of parameter values. DLAMOs are predicted to occur in the region between these two curves of Hopf points. The bifurcation curves meet at the point (*g_KCa_* = 0.4 nS, *b_s_* = 5.5e-3 mM/ms). Thus, DLAMOs are not expected in cells with very high basal calcium or very low K_Ca_ channel expression. Raising extracellular Ca^2+^, or pharmacologically blocking/opening K_Ca_ channels, may induce or inhibit DLAMOs by effectively shifting the cell to a different region in this parameter plane.
(TIF)

Figure S3 One-parameter bifurcation diagram of the model's behavior as a function of basal shell calcium for low K_Ca_ conductance (*g_KCa_* = 0.1 nS). As *b_s_* is increased from zero, the depolarized steady state (upper thick black line) disappears at a saddle-node bifurcation (LP_1_), causing an abrupt transition to a hyperpolarized steady state (lower thick black line). If *b_s_* is then decreased from this point, the model exhibits hysteresis and stays at the hyperpolarized steady state until it disappears via saddle-node bifurcation at LP_2_. Thus, for *b_s_* between LP_1_ and LP_2_, the model is bistable.
(TIF)

Figure S4 Two-parameter bifurcation diagram showing the location of the saddle-node bifurcation points in Figure S3 (LP_1_ and LP_2_) as basal shell calcium and *g_KCa_* are varied simultaneously. For *g_KCa_* values below 0.015 nS, no bifurcations occur and the model has a single stable steady state for all values of *b_s_*.
(TIF)

Figure S5 Membrane excitability promotes gene expression rhythms in the absence of sodium-dependent AP firing. Simulations of the extended gene regulation model of Figure 6C with hyperexcited states (top) leading to peaks in calcium concentration (middle) and mRNA level (bottom) as shown in Figure 6D. At *t* = 84 hours, application of TTX was simulated by setting *g_Na_* = 0. Although the model no longer fires APs, the ∼24-hour oscillations in cytosolic calcium and clock gene expression persist.
(TIF)

Figure S6 Visualization of calcium dynamics. Model fires APs (top left) upon release from a hyperpolarizing current (Iapp = −5 pA), leading to an influx of calcium current (bottom left) and increases in calcium concentration in the shell (top right) and cytosolic (bottom right) compartments.
(TIF)
