# Supplementary figures and images for: Causes and Consequences of Hyperexcitation in Central Clock Neurons
Source: PLoS Comput Biol. 2013 Aug 22;9(8):e1003196. doi: 10.1371/journal.pcbi.1003196 (PMC3749949; doi:10.1371/journal.pcbi.1003196)

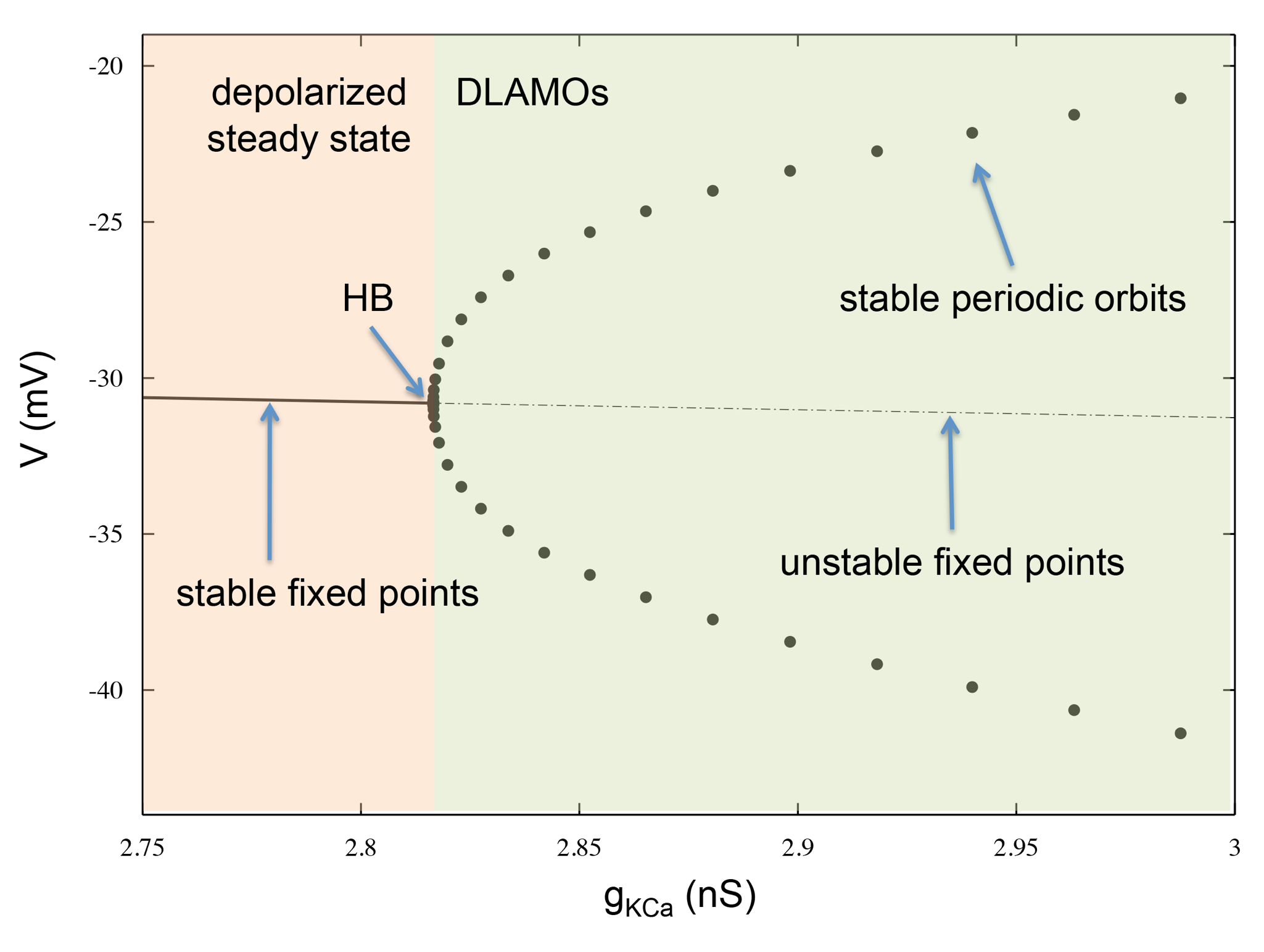

Supplement: Figure S1 — One-parameter bifurcation diagram of the model's behavior as a function of KCa conductance. For a given basal calcium concentration in the shell (here bs = 5.425e-4 mM/ms), the depolarized steady state (solid black line) becomes unstable (dashed black line) through supercritical Hopf bifurcation (HB at gKCa = 2.82 nS, V = −30.8 mV) as gKCa is increased. At the HB point, stable periodic solutions modeling DLAMOs are born (circles indicate maximum and minimum voltage values for the oscillations at various gKCa values). The regions of gKCa corresponding to the depolarized steady state and to DLAMOS are shaded light red and light green respectively. The depolarized steady state persists for gKCa values below 2.75 nS. (TIF) [file pcbi.1003196.s001.tif]

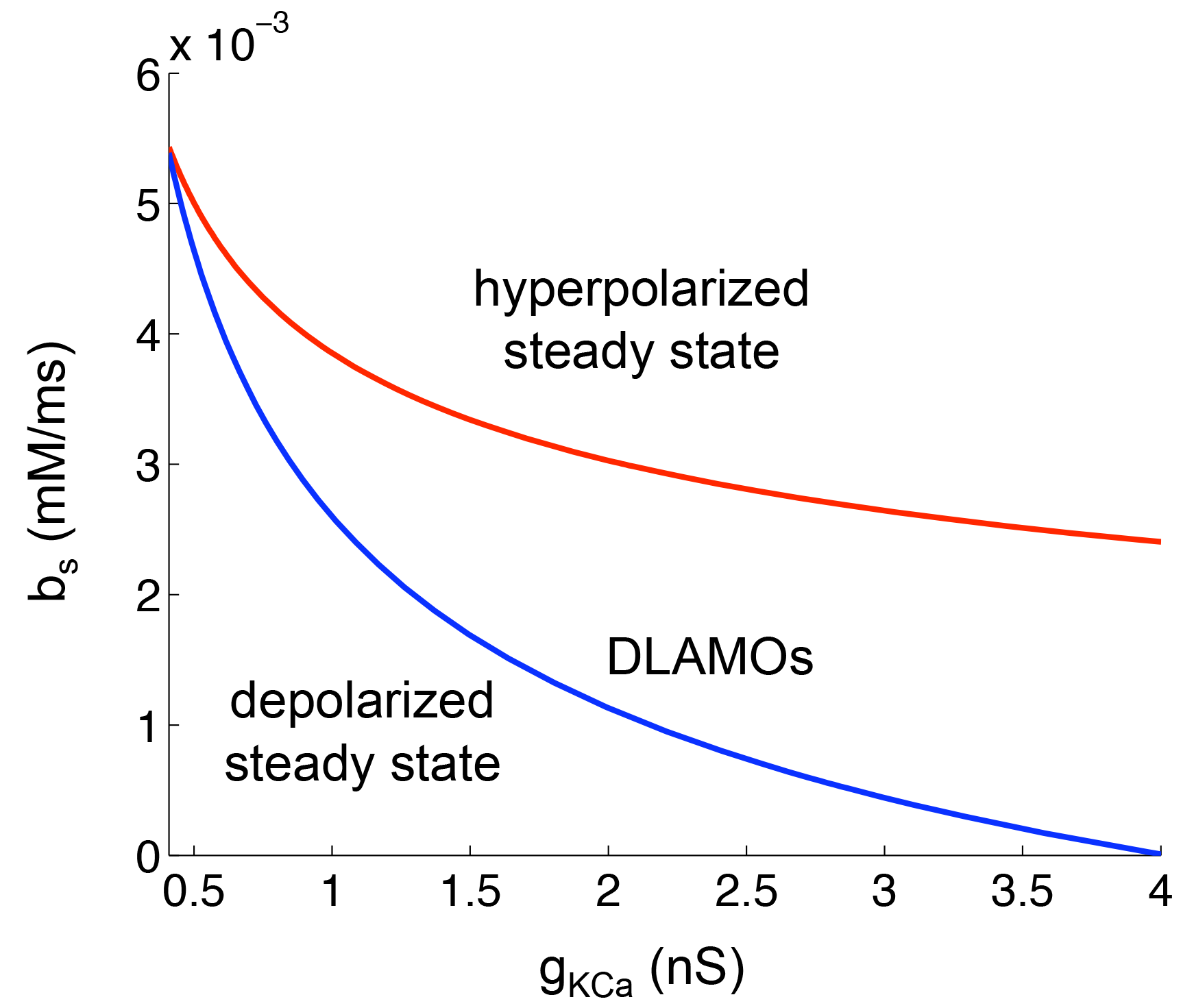

Supplement: Figure S2 — Two-parameter bifurcation diagram showing the location of the Hopf bifurcation point in Figure S1 (blue line) as a function of gKCa and basal calcium concentration in the shell. As bs increases, less KCa conductance is required to undergo bifurcation from the depolarized steady state to DLAMOs. A second Hopf bifurcation (red line) from a hyperpolarized steady state occurs at certain combinations of parameter values. DLAMOs are predicted to occur in the region between these two curves of Hopf points. The bifurcation curves meet at the point (gKCa = 0.4 nS, bs = 5.5e-3 mM/ms). Thus, DLAMOs are not expected in cells with very high basal calcium or very low KCa channel expression. Raising extracellular Ca2+, or pharmacologically blocking/opening KCa channels, may induce or inhibit DLAMOs by effectively shifting the cell to a different region in this parameter plane. (TIF) [file pcbi.1003196.s002.tif]

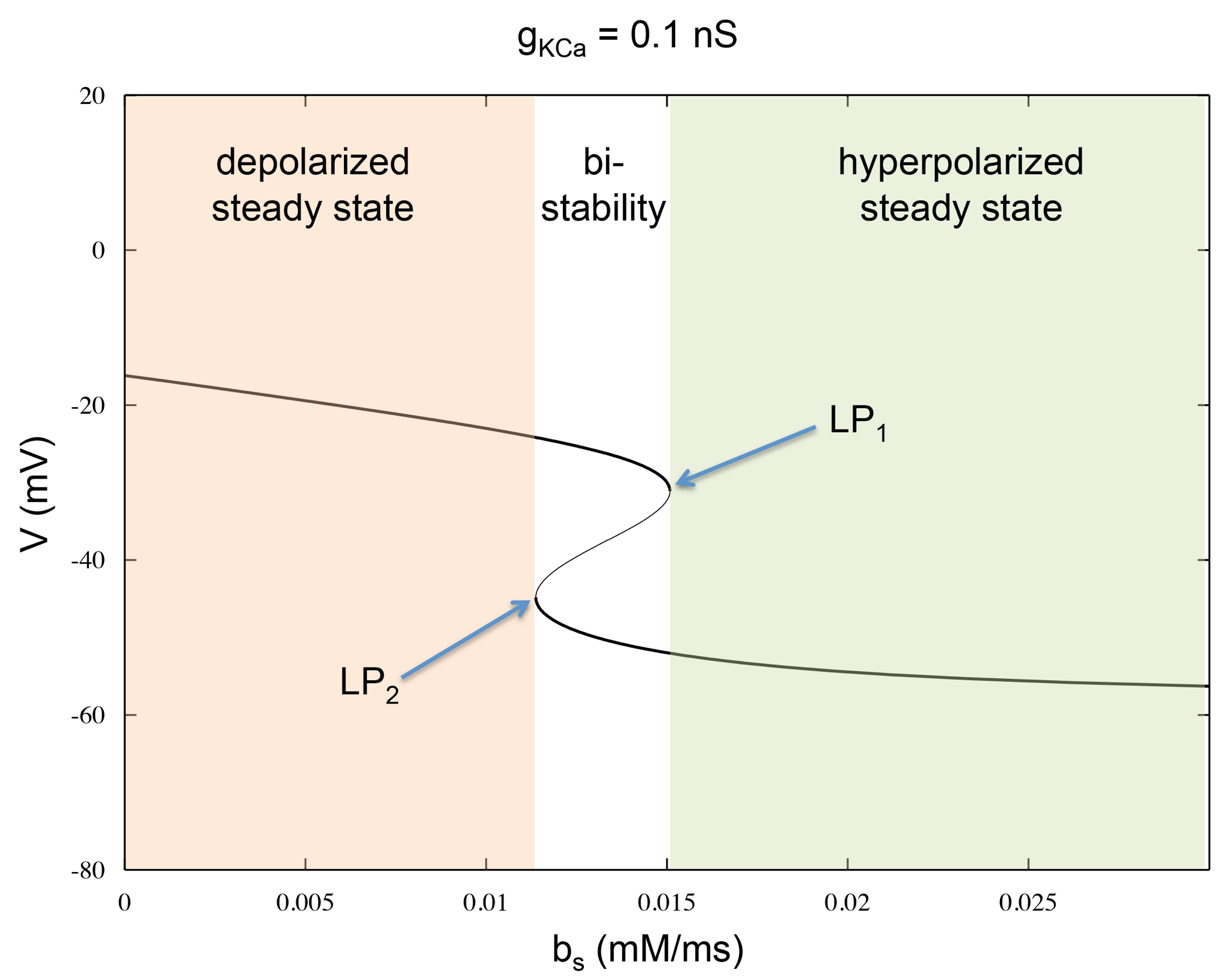

Supplement: Figure S3 — One-parameter bifurcation diagram of the model's behavior as a function of basal shell calcium for low KCa conductance (gKCa = 0.1 nS). As bs is increased from zero, the depolarized steady state (upper thick black line) disappears at a saddle-node bifurcation (LP1), causing an abrupt transition to a hyperpolarized steady state (lower thick black line). If bs is then decreased from this point, the model exhibits hysteresis and stays at the hyperpolarized steady state until it disappears via saddle-node bifurcation at LP2. Thus, for bs between LP1 and LP2, the model is bistable. (TIF) [file pcbi.1003196.s003.tif]

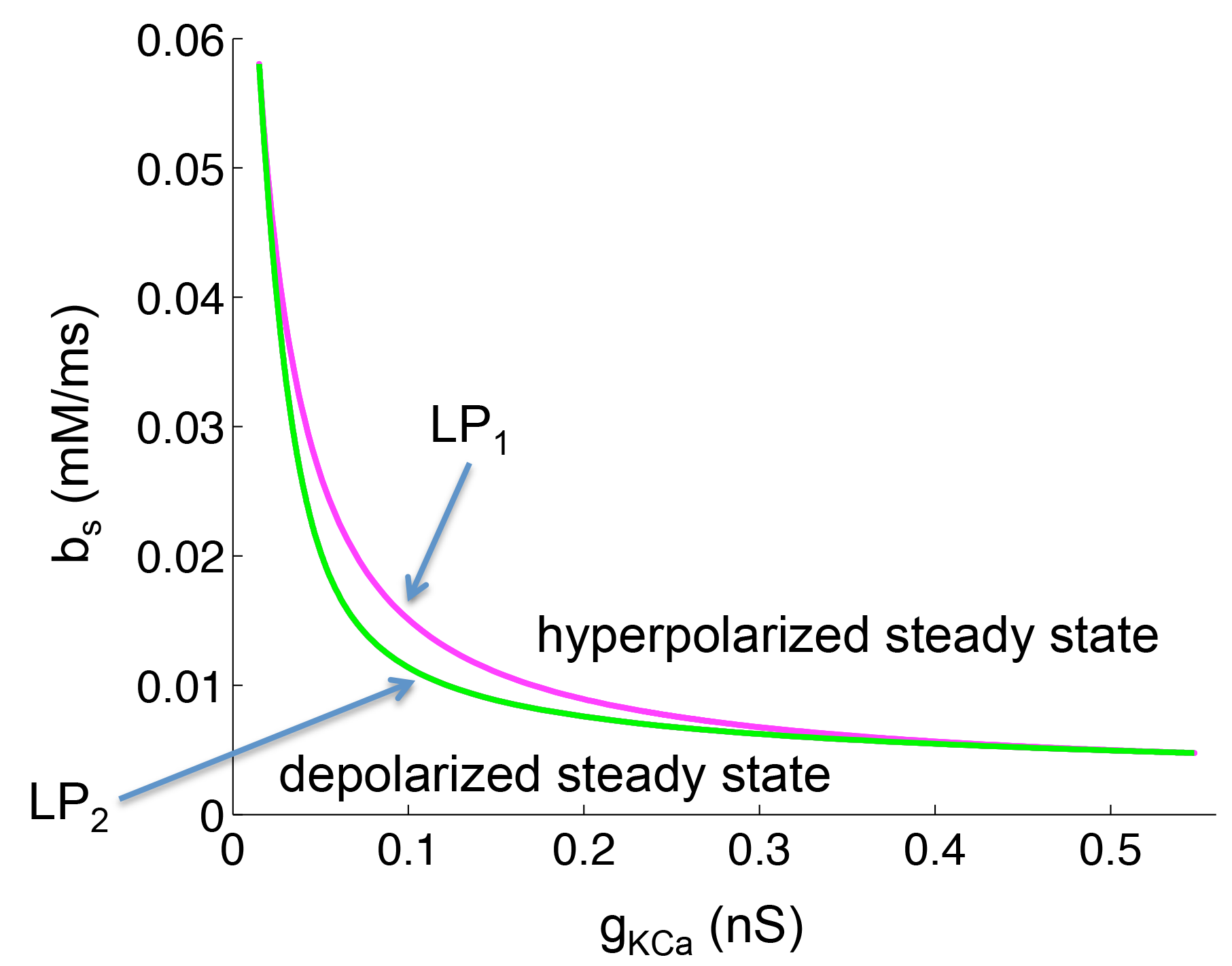

Supplement: Figure S4 — Two-parameter bifurcation diagram showing the location of the saddle-node bifurcation points in Figure S3 (LP1 and LP2) as basal shell calcium and gKCa are varied simultaneously. For gKCa values below 0.015 nS, no bifurcations occur and the model has a single stable steady state for all values of bs. (TIF) [file pcbi.1003196.s004.tif]

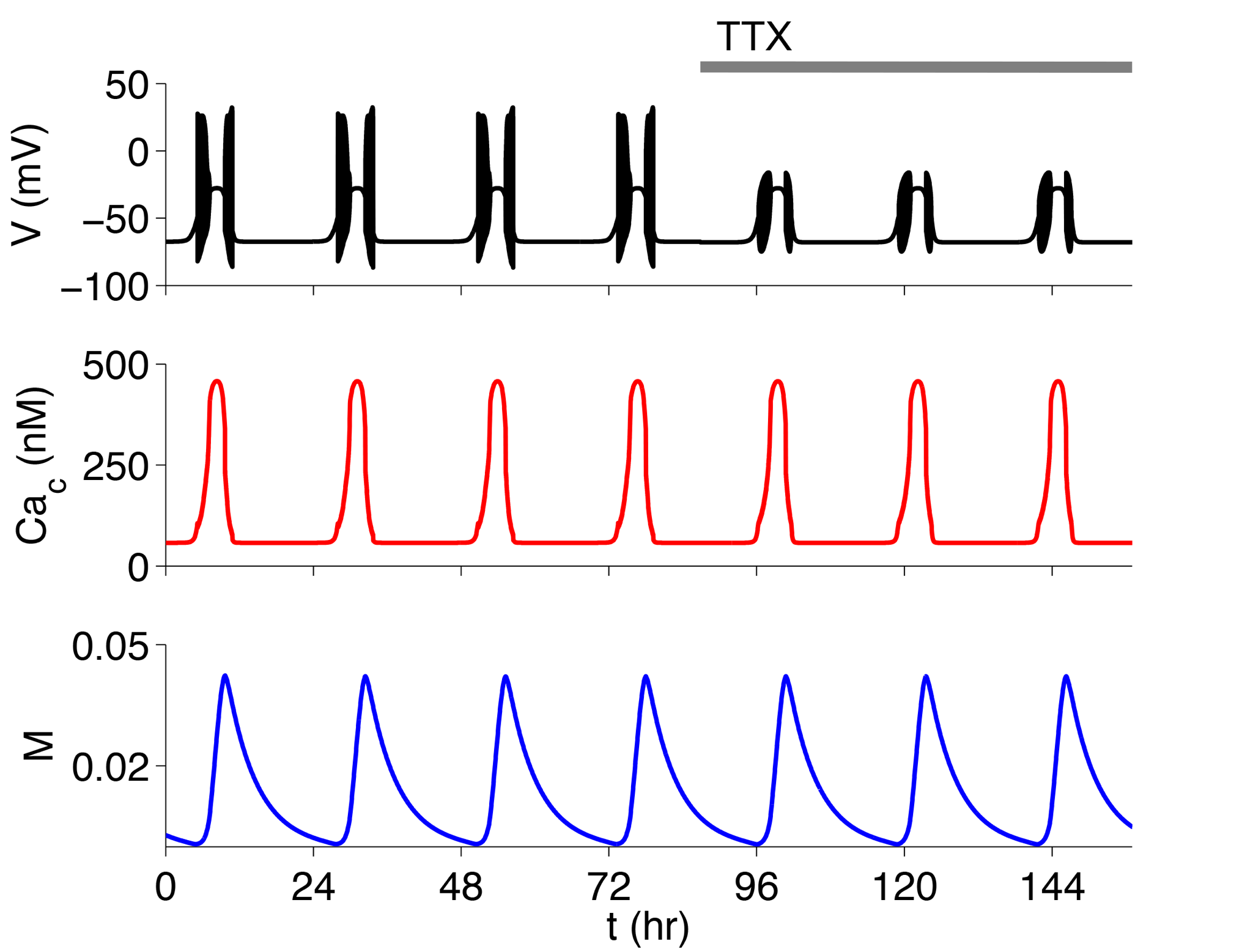

Supplement: Figure S5 — Membrane excitability promotes gene expression rhythms in the absence of sodium-dependent AP firing. Simulations of the extended gene regulation model of Figure 6C with hyperexcited states (top) leading to peaks in calcium concentration (middle) and mRNA level (bottom) as shown in Figure 6D. At t = 84 hours, application of TTX was simulated by setting gNa = 0. Although the model no longer fires APs, the ∼24-hour oscillations in cytosolic calcium and clock gene expression persist. (TIF) [file pcbi.1003196.s005.tif]

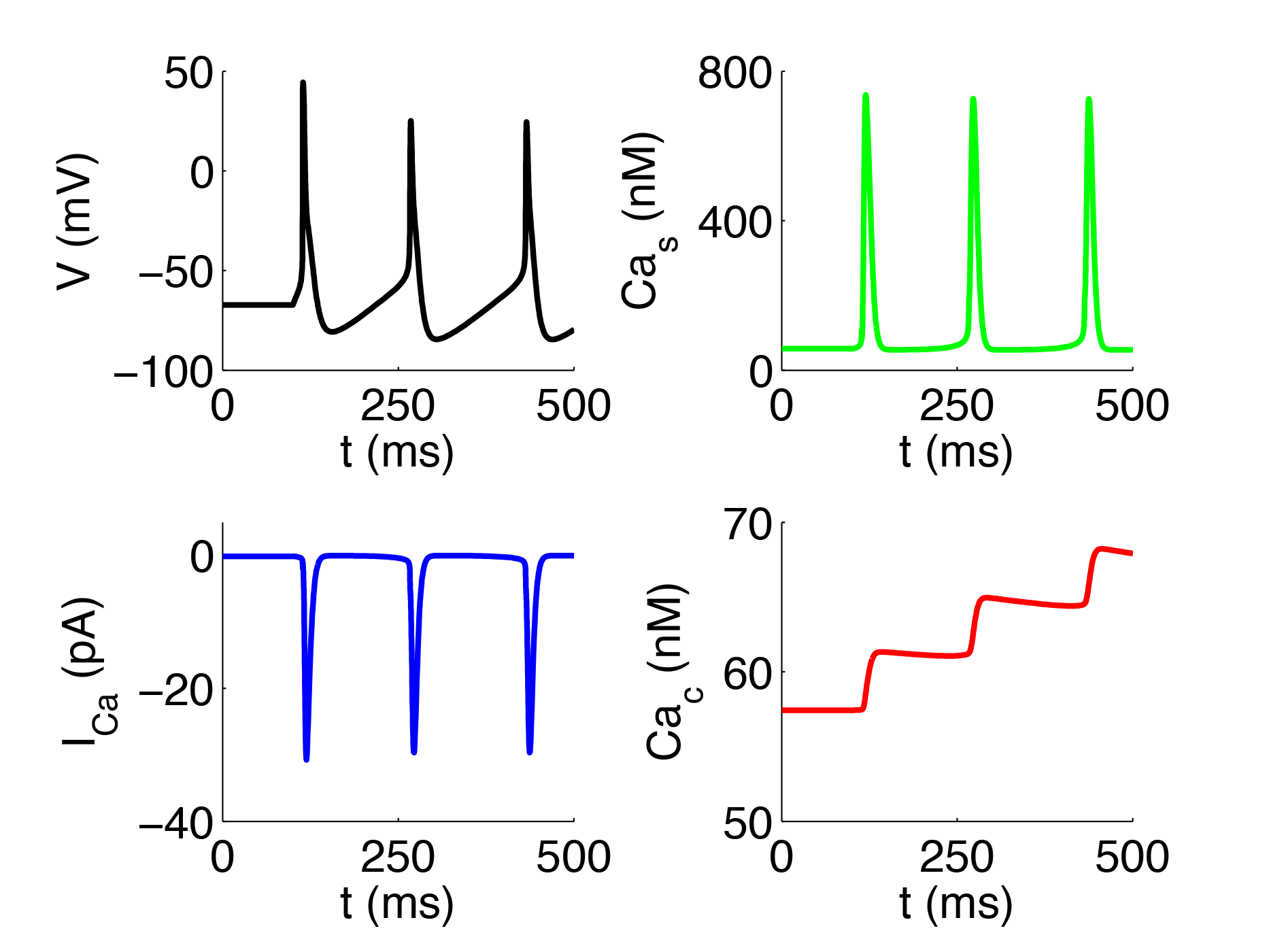

Supplement: Figure S6 — Visualization of calcium dynamics. Model fires APs (top left) upon release from a hyperpolarizing current (Iapp = −5 pA), leading to an influx of calcium current (bottom left) and increases in calcium concentration in the shell (top right) and cytosolic (bottom right) compartments. (TIF) [file pcbi.1003196.s006.tif]
